# Physical activity behaviours of Culturally and Linguistically Diverse (CALD) women living in Australia: A qualitative study of socio-cultural influences

**DOI:** 10.1186/1471-2458-11-26

**Published:** 2011-01-11

**Authors:** Cristina M Caperchione, Gregory S Kolt, Rebeka Tennent, W Kerry Mummery

**Affiliations:** 1 Faculty of Health and Social Development, University of British Columbia, Kelowna, Canada; 2 School of Biomedical and Health Sciences, University of Western Sydney, Sydney, Australia; 3 Research School of Social Sciences, The Australian National University, Canberra, Australia; 4 Faculty of Physical Education and Recreation, University of Alberta, Edmonton, Canada

## Abstract

**Background:**

Australia continues to witness rising levels of immigration by individuals from Culturally and Linguistically Diverse (CALD) origins. With this rapidly growing diverse population, Australia faces a number of population health challenges. In particular, CALD women have been shown to be at an increased risk of chronic diseases such as cardiovascular disease, diabetes, and poor mental health. Despite the high risk of these diseases, women from CALD groups are less likely to be proactive in accessing health care or undertaking preventative behaviours, such as physical activity participation. The purpose of this study was to examine the socio-cultural influences on the physical activity behaviours of CALD women living in Australia by identifing the barriers, constraints and possible enablers to physical activity participation for this population.

**Methods:**

Twelve focus group sessions were undertaken with CALD women (N = 110) from Bosnian, Arabic speaking, Filipino and Sudanese communities in three regions: New South Wales, Victoria, and Queensland. In a semi-structured, open table discussion, participants were encouraged to share their opinions, perceptions and beliefs regarding socio-cultural influences on their physical activity behaviours. Common and ethnic-specific themes emerged from the discussions.

**Results:**

Common themes included: knowledge of physical activity, differing physical activity levels, and the effects of psychological and socio-cultural factors, environmental factors, and perceptions of ill-health and injury, on physical activity behaviours. Ethnic-specific themes indicated that post-war trauma, religious beliefs and obligations, socio-economic status, social isolation and the acceptance of traditional cultural activities, greatly influenced the physical activity behaviours of Bosnian, Arabic speaking, Filipino and Sudanese women living in communities throughout Australia.

**Conclusions:**

This study demonstrates that attitudes and understandings of health and wellbeing are complex, and have a strong socio-cultural influence. The findings of the present study can be used not only to inform further health promotion initiatives, but also as a platform for further research with consumers of these services and with those who deliver such services.

## Background

Australia has witnessed a rapid increase in migration over the past 10 years, with annual migrant numbers doubling as a proportion of the total population [1]. With the diversity of this growing population Australia faces a number of population health challenges. Close examination of epidemiological data reveals particular burdens of disease in women from Culturally and Linguistically Diverse (CALD) communities now living throughout Australia [2]. Moreover, there is a consensus among western countries, including Australia, New Zealand, United Kingdom, United States and Canada, that significant racial and ethnic disparities exist with regard to prevalence, mortality, and morbidity, highlighting higher rates of risk factors for a number of chronic diseases in these women [3,4]. Of particular concern is the greater risk of hypertension, diabetes, and overweight/obesity, all of which are predominant risk factors to cardiovascular disease (CVD) [4,5].

Despite the high risk of these non-communicable diseases, women from CALD groups are less likely to be proactive in accessing health care or undertaking preventative measures to reduce risk of chronic disease [6,7] and ensure optimal health outcomes [8,9]. Physical activity, which is operationally defined as any bodily movement produced by skeletal muscles that results in energy expenditure [10], is a particular preventative measure in which women from CALD backgrounds are less likely to undertake then other non-CALD women [11,12]. For instance, CALD women born outside of Australia have reported a 20% less participation rate in sport and physical activity in Australia compared to Australian born women (46.3% in CALD women compared to 66.5% in Australian born women) [12].

For many CALD individuals there are several constraints on activity participation beyond personal motivation. Cultural barriers, socioeconomic factors, psychological trauma relating to migration, perceptions of ill health and injury, and alternate health seeking behaviours are just a few of the constraints that are likely to have a detrimental impact on health in these populations [13-16]. In an attempt to limit these constraints and positively influence the physical activity behaviours of CALD women, it is necessary to carefully consider cultural diversity whilst developing and planning health promotion (e.g., physical activity) resources and programs. The limited nature of research in this area is evident [2,17,18]. Despite the significance of the work outlined above, there are a number of gaps in the literature. Thus, the purpose of this study was to build upon existing, yet limited research, and examine the socio-cultural influences on the physical activity behaviours of CALD women living in Australia by identifing the barriers, constraints and possible enablers to physical activity participation for this population. Such information will support the development of culturally appropriate programs designed to positively influence the physical activity behaviours of women from CALD populations [14,19].

## Methods

### Study Participants

This study was conducted with CALD women living in three Australian cities with the highest proportion of CALD migrants: Sydney, New South Wales; Brisbane, Queensland; and Melbourne, Victoria [20]. Four distinct CALD groups were chosen as they were identified as being interested in participating, and were accessible to the research team due to prior research linkages the researchers had with organisations that provided services for these groups. These included Bosnian, Arabic speaking (including women from Eygpt, Iraq, Syria, Jordan, Palestine, and Lebanon), Fillipino, and Sudanense adult women (18 years+). A total of 110 women participated in one of 12 focus group sessions, separated by CALD group. Mean age was 46.2 (SD ± 11.6) years, with an age range of 18-87 years. The majority of the women were married and had children. All participants were born outside of Australia and had lived in Australia for an average of 12.4 years. Participant characteristics are detailed in Table 1.

**Table 1:** Demographic Characteristics of Study Participants

Variable	Participants (N = 110)
Age of each group (Mean ± SD)	
Filipino	55.7 (15.3)
Sudanese	26.8 (7.0)
Bosnian	63.0 (13.1)
Arabic Speaking	39.1 (10.4)
	
Cultural group (%)	
Filipino	28.2
Sudanese	22.7
Bosnian	22.7
Arabic Speaking	26.4
	
Years living in Australia (Mean ± SD)	
Filipino	18.2 (9.1)
Sudanese	5.0 (2.4)
Bosnian	14.8 (10.2)
Arabic Speaking	11.6 (9.8)
	
Marital Status (%)	
Married	81.0
Divorced	2.0
Widowed	7.0
Not married	10.0
	
Total those with children (%)	86.0
Children per woman (Mean ± SD)	
Filipino	3.1 (1.7)
Sudanese	1.7 (1.1)
Bosnian	2.5 (1.1)
Arabic Speaking	3.1 (1.4)

### Study Design and Procedures

The proposed project is exploratory in nature and aims to explore perceptions, attitudes, opinions, and beliefs concerning the physical activity behaviours of CALD women. With this intention, the researchers chose to utilise focus group research as a method of data collection. Focus group research is recognised as an exploratory research method that best draws upon respondents' attitudes, feelings, beliefs, experiences and reactions in a way in which would not be feasible using other methods, for example, one-to-one interviewing [21]. These attitudes, feelings and beliefs may be partially independent of a group or its social setting, but are more likely to be revealed via the social gathering and the interaction which being in a focus group entails.

With assistance from community partners in each of the three cities, CALD women were recruited to take part in focus group sessions between April and July, 2009. CALD women who were clients, accessed services, or who were somehow associated with each community partner organisation were contacted by phone, email or in person by a community health worker or project officer from each partner organisation. These partnerships were a crucial aspect of the overall project as each partner provided comprehensive knowledge and advice pertaining to the different CALD groups, as well as playing a lead role in recruiting participants. Each of these community partners are not-for-profit organisations funded by different arms of their own state government (e.g. health services, immigration services). These community partners represent the interests of the many CALD groups throughout New South Wales, Queensland, and Victoria, particularly focusing on such matters as health promotion, cultural diversity and health, cultural competence, language services, cross cultural communication, health assessment and consumer participation.

A total of 125 potential participants were contacted and invited to participate in the focus group sessions. Initially, all 125 invited participants agreed to take part in the project, however only 110 participants attended each of their CALD specific focus groups. Participants who did not attend the groups (N = 15) indicated that they had either forgotten about the focus group session or had an unplanned matter that they had to attend to. Researchers from the project team undertook four focus group sessions (one representing each of the four CALD groups) in each of the three cities (total of 12 focus groups). These included Bosnian (N = 25), Arabic speaking (N = 29), Fillipino (N = 31), and (4) Sudanense (N = 25) women. Participation was voluntary, however all participants were given a $40 honorarium to assist with transport and childcare costs for the duration of their focus group session.

The principal researcher (CC) acted as the moderator, guiding the discussion and providing assistance where needed, whilst a second researcher (RT) took notes and was responsible for the audio recording of each session. A translator/interpreter was used during four focus group sessions (one Arabic group and all three Bosnian groups) to accommodate CALD group participants who spoke minimal or no English. Although these sessions did take longer to complete, the researchers felt that these sessions flowed well and were not disrupted by the language barrier. All other sessions were held in English at the request of the group members. Focus groups were held at a central location convenient for participants and ranged from 45-90 minutes in duration. During this time participants were encouraged to share their opinions, perceptions and beliefs regarding the barriers and enablers to women's physical activity, in a semi-structured, open table discussion.

Questions in the focus group schedule were guided by the objectives of the project and based on previous literature concerning the physical activity and health behaviours of women [7] and CALD populations [9]. As well, the focus group schedule was informed by questions used in previous studies by members of the research team [22-24]. Questions were open-ended in order to encourage a range of responses, and probes and clarifying questions were used to stimulate further discussion. For example, to uncover some of the barriers and motives to physical activity participation in CALD groups, participants were asked 'Can you tell me some of the reasons why you and other people from your community might *not *participate in physical activity?' and 'What are some of the things and/or reasons that would motivate you to regularly participate in physical activity?'.

### Data Management and Analysis

Following each of the sessions the data were professionally transcribed verbatim. QSR NVivo qualitative analysis software (QSR International Pty Ltd, Melbourne, Australia) was used to organise and manage the data. Using an inductive approach, data analysis focused on eliciting themes concerning barriers and enablers to women's physical activity participation. Research team members systematically read the transcripts multiple times, highlighted segments of interest and made annotated comments on the transcripts to identify potential themes. Emerging themes were summarised and categorised during the process of reading and rereading. Structural corroboration, where segments of data validated each other, was performed by noting emerging descriptors, issues and concerns in each transcript [25]. Members of the research team reached consensus concerning emerging themes and categories through a process of ongoing discussion to mutually resolve any discrepancies or concerns with analysis. Final themes and categories were identified by the researchers and are summarised in the results section below.

Ethical approval was obtained from the Central Queensland University's Human Research Ethics Committee. The standard university guidelines of informed consent, voluntary participation, confidentiality, and anonymity were rigorously followed. All participants gave written and verbal informed consent prior to each focus group session.

## Results

The themes identified are reported in two ways: first, the themes common to all CALD groups are reported, and second, those relevant to specific CALD groups are discussed.

### Common themes

#### Knowledge and Awareness of Physical Activity

The responses from most participants indicated that they saw physical activity in the broad context that covered a range of activities usually aligned with Western interpretation of physical activity. Participants were also able to distinguish between moderate and vigorous physical activity. This is demonstrated by, physical activities such as walking, gardening and stretching being suggested by participants as examples of 'moderate physical activity', whilst participants tended to refer to 'sport' as vigorous exercise (defined as a subset of physical activity that is planned, structured, and repetitive and has a final or intermediate objective, the improvement or maintenance of physical fitness [10]). This included activities such as jogging and swimming. Most participants described daily activities such as sweeping, vacuuming and washing clothes as physical activities, however a small number of participants were unsure whether such incidental activities were considered as physical activities. These participants suggested that such home duties were traditionally just part of the normal day, indicating that

*Housework is just working everyday, I would not say it is sport or exercise, I feel we have to make special exercises for our bodies*. (Filipino participant)

Although some of the participants engaged in leisure or recreational type physical activity, most of the physical activity undertaken by participants originated from daily incidental activity or walking for transport. Furthermore, when discussing physical activity the majority of participants used the terms physical activity and exercise interchangeably, suggesting that they were not clear on how each differed from one another.

#### Levels of Physical Activity Change Upon Migration

In general, participants were more active in their country of origin than in Australia due to the more physical nature of everyday life. For example, many suggested that in their birth country there was less reliance on cars; water was derived through water pumps located some distance from the home; homes had bare floors that required sweeping daily; and there was less access to labour saving devices such as vacuum cleaners and washing machines. One respondent summarised the words of many:

*In Egypt it was completely different. What I saw is that they all live in apartments, so they all take the stairs up and down. There's barely any lifts. And if there are lifts they don't normally work. They walk to the markets. Not many people have cars so they walk everywhere. Even if they take the bus, they walk around the shopping centres cause most of them are not malls. They're outside shopping centres and you walk from store to store*. (Arabic speaking participant)

#### Psychological and Socio-cultural Factors

The vast majority of participants indicated that family commitments frequently prevented them from undertaking physical activity. Most undertook the bulk of the domestic duties; this was suggested to be the cultural norm for each of the groups. The following quote resonates with the majority of responses:

*Everyone has different lives. Sometimes I have to start work at 8:30. I leave so early for the traffic. Then I finish 3:30, 4:00, and when I reach home I have to cook for my family. After dinner we have to clean up, get ready for bed, for the next day. Tell me when I am going to do exercise*. (Bosnian respondent)

While several participants indicated that having young children led them to undertake more physical activity, such as dancing and walking, no participants linked physical activity to their own health or the health of their family. Participants understood that being physically active was beneficial for health and wellbeing, yet they did not recognise it as a important daily necessity, but rather a luxury.

#### Environmental Factors

Safety was a key concern for the women as many lived in areas with higher than average crime rates and thus found it difficult to feel safe and at ease in their new country. In most cases, women would only undertake physical activity outside of the home during daylight hours. In a couple of cases, women reported having been followed by strangers:

*I always set aside the time for me to walk in the morning but then one time I was walking, there was somebody who stopped his car near me and followed me. I didn't go walking any more after that*. (Filipino respondent)

Most participants acknowledged that physical activity could be undertaken in and around the home for free; however they were less likely to be motivated without the support of a group.

A further problem was in knowing where to access information about community activities, assuming that programs did exist. Many of the participants were unaware if any physical activity initiatives actually existed in their local community, and if they were aware of them, many did not know where or how to access such programs or initiatives.

#### Perceptions of Ill-Health and Injury

The health concerns of participants were both a motivator and a limitation to participating in physical activity. Some participants commenced new and different physical activities due to medical concerns. In this regard, health "scares" was often a motivating factor for increasing physical activity. Conversely, participants indicated that too much physical activity could create perceived health concerns such as injury, tiredness and soreness. As one respondent said, "My mother told me, remember your uterus will drop if you do physical activity" (Filipino respondent). Yet others were limited in the physical activity they could undertake due to illnesses. For example:

*I have problems in my cervix, so I feel restricted to the kind of exercise I can handle... I used to love to swim but I started to get cramps so now I panic when I am swimming*. (Arabic speaking respondent)

Many of the participants were unclear as to what constitutes health, and/or had different understandings of the association between health and physical activity. For example, one respondent explained that weight loss associated with being physically active was considered negative; it was understood that being overweight was a sign of health, prosperity and happiness.

*In Africa we have this belief, the bigger you are the better. You are rich, you are happy and healthy. You are skinny they think you are sick. I think someone skinny in Africa has AIDS*. (Sudanese respondent)

Different understandings of health and the association between health and physical activity were common amongst the majority of the CALD groups.

### Ethnic-specific themes

#### Bosnian

Most of the women in this group were suffering from post-war trauma, and suggested that in general, they were not coping with life well in Australia. This had led to lack of motivation for physical activity, as well as experiences of depression, stress and overeating. In the words of one respondent:

*I just think one of the main points is that we have abnormal circumstances (due to post-war stress and post-war trauma). We are not coping good now. Even if we are the same age, a 50 year old woman in Bosnia and a 50 year old woman in Australia is not the same. Our circumstances have made us more depressed [than Australian women]. If you are depressed you are not able to socialise. If you are not able to socialise you are eating more*. (Bosnian respondent)

Nonetheless, for several participants trauma was reported to be a motivator for physical activity, on the basis that they believed physical activity to promote psychological wellbeing.

By comparison to the Arabic speaking group, which similarly comprised a majority of Muslim women, the Bosnian participants suggested that being physically active was a religious obligation, although all conceded that they needed to do more physical activity. Religious adherence was not considered to bring about any barriers to physical activity. In fact, this group considered prayer to be a form of physical activity.

#### Arabic-speaking

A key theme for Arabic-speaking women was finding the time to be physically active. This group suggested they had less time for physical activity due to larger than average family sizes and cultural norms that required the bulk of normal domestic duties, including cooking, cleaning and child care, to be undertaken by women. This was assumed regardless of external employment. One respondent summarised the experiences of many:

*In our culture men usually do nothing at home. Women do the cooking and the cleaning, even if they work. If they want to do something they don't have the chance*. (Arabic speaking respondent)

The need for public modesty for women was also identified as a barrier to physical activity outside of the home. For this reason, the group stipulated the need for formal indoor facilities where activities such as swimming and gym activities could be undertaken in the presence of women only:

If we would like to go to the gym then we have to mention the person there, there is no men to go inside. That's a bit of problem for us. You have to trust the people there, because it's our religion. It's very hard.

Many women suggested that local indoor pools with women-only times would be useful. With a strong cultural emphasis on family, the ideal facility for this group was an internally-segregated centre where men, women and children could attend at the same time. It was thought that this type of facility would be beneficial in promoting increased physical activity.

#### Filipino

Socio-economic status played an important role in determining changes in physical activity upon migration for those born in the Philippines. Those with government or other office jobs in the Philippines were more likely to have similar levels of physical activity before and after migration. For example, these participants tended to use private transport in the Philippines, visit a gym for physical activity and have less-laborious domestic duties. Other Filipino women suggested that migration resulted in significantly different lifestyles, undertaking less physical activity due to increased reliance on private transport, domestic labour saving devices and in-house access to water. Participants also suggested that those who lived in rural areas tended to be more active than those living in cities; this impacted upon changes in physical activity after migration to Australia. A small number of participants said they were more physically active in Australia than they were in the Philippines because they walked more in Australia. Similar to other CALD groups, a key issue was lack of time to partake in physical activities. One respondent highlighted this problem:

*In Australia, we don't have any help at all. In our country, we have our relatives, like brother or sister or nephews or nieces to look after the kids while we can have time a little bit to ourselves to relax. But in here you tend to do everything and as they said, it's more kind of a stressful way financially and physically*. (Filipino respondent)

#### Sudanese

Sudanese women in this study reported childcare and other home duties as significant barriers to undertaking more physical activity. Many lacked support of family and friends in Australia, and suggested they were socially isolated.

A lack of understanding within Australia of Sudanese cultural norms also played a role in preventing Sudanese women from being active. The women mentioned that they were reluctant to undertake common forms of physical activity, due to these misunderstandings. For example, these women outlined how they would normally carry their children tied onto their backs (as a physical activity) to help maintain their strength, but had been prevented from doing so in Australia. Specifically, one woman recalled a time where a friend was questioned by local police officers while undertaking such an activity in her own neighbourhood.

*In Sudan we used to carry our child [on our back]. Every woman have a child, put child [on the back] and run... But my friend [here in Australia], she tied her kid on with a rock pile and she got in trouble with the cops. They thought she was trying to steal her child. Now we don't have this way to carry. So less exercise*. (Sudanese respondent)

Similar to the Bosnian women, barriers to physical activity were also psychological in nature; many participants had migrated to Australia as the result of war in Sudan, and this led to stress and a lack of motivation for physical activity. As one respondent highlighted:

Such things are traumatic... it helps with issues of stress but when you're tired and stressed, you're lazy. I know that when you run, you release that stress...

## Discussion

This study revealed a number of common and ethnic-specific themes concerning the physical activity behaviours of Bosnian, Arabic speaking, Filipino and Sudanese women. With regards to common themes, most participants described what physical activity was within a broad context (and in line with a Western interpretation), and were able to distinguish between moderate and vigorous intensity physical activity. This is largely inconsistent with previous research which has indicated that the use of these modifiers (moderate, vigorous) are commonly misunderstood by the mainstream white culture and further complicated when culture and language translations are considered [26]. A clear understanding of what physical activity is and how much is good for you may be influenced by the increase in public health messages regarding recommended levels and intensities of physical activity.

When discussing physical activity levels at the present time and prior to migration to Australia, the majority of participants indicated that they were much more active in their country of origin due to the more labour-intensive nature of daily life. These responses were consistent with what researchers have labelled the 'healthy immigrant effect'. A 'healthy immigrant effect' exists where migrants are in generally very good health on arrival to a western country, however, this condition changes with increased time since migration, and is associated with acculturation, defined as changes in cultural patterns when groups of individuals from different cultures come into first-hand continuous contact with each other [27-29]. Acculturation is often associated with detrimental behaviours such as the consumption of high fat, calorie dense diets and physical inactivity [30]. It has been suggested that an educational component should be a major part of any health promotion initiative, paying close attention to cultural differences pertaining to the interpretation, and benefits of, physical activity [31].

The majority of participants indicated that family commitments, in particular childcare and domestic home duties, such as preparing meals and cleaning the home, prevented them from being physically active. This is a common theme between both CALD women and Australian born women who have indicated that finding time to be active outside of their family duties is a major barrier [31,32]. It has been suggested that competing demands may only be constraints for organised activities that require women to attend activity sessions at a specific time and place [33,34]. Encouraging women to engage in physical activities that fit into the context of their daily lives may help to overcome this barrier [31].

Environmental factors such as safety concerns and access to programs and facilities were reported as common barriers to physical activity for CALD women. Structural environmental changes (e.g. improved lighting, well maintained footpaths, access to indoor facilities, etc.) have been reported as a way to overcome safety issues as well as assist with facility accessibility. In addition, offering programs and facilities in the heart of these local neighbourhoods will increase program awareness and allow for these facilities to be easily accessed. This has become a common practice in the United Kingdom in which local health organisations and centres have set up 'health action zones' in deprived communities where many CALD communities reside [35]. These 'health action zones' provide health care resources and different health promotion initiatives in an area of the community in which the majority of community members can walk to. Similar zones should be considered in specific suburbs of Sydney, Melbourne and Brisbane, and other areas which are heavily populated by CALD communities.

Ill health and injury was both a motivator and barrier to participants' physical activity engagement. Consistent with the Health Belief Model [36], some participants perceived their ill health to be at a level of severity that required action, while others were fearful of being susceptible to disease due to an unhealthy lifestyle. When the risk of disease, and possibly death, is described as a consequence of physical inactivity and an unhealthy lifestyle, women in particular are motivated to change their lifestyle behaviours when they realise the benefits of doing so [37]. This is synonymous with the findings of the current study, with a number of older participants making changes to their lifestyle behaviours by attempting to be more physically active and making healthier diet choices.

Ill health and injury also acted as a barrier to physical activity for many participants. Specific to CALD populations, perceptions of ill health and injury associated with being physically inactive may be related to different understandings and/or misunderstandings of physical activity and its' benefits. For instance, previous research has reported that many Arabic speaking groups perceived sweating, increased heart rate, and breathlessness as illness states rather than 'normal' by-products of physical activity [14]. Similarly, many of the participants in our study also associated physical activity with health concerns such as injury, tiredness and soreness. Although these different understandings may be conflicting with the health and physical activity messages traditionally promoted in Australia, they are not necessarily incorrect or inappropriate. It is essential that health professionals are sensitive to the different understandings and perceptions that some CALD groups may have regarding health and physical activity, as many CALD groups believe that their understanding of physical activity and health is both culturally appropriate and legitimate.

In addition to the common themes outlined above, there were also a number of ethnic specific themes revealed by each of the four groups. For example, Bosnian women highlighted the detrimental effect of the experiences of war, indicating that depression and stress are common symptoms of post-war trauma, and that these psychological states limit their motivation to be active. Post-war trauma and the effect it has on health, has commonly been associated with migrant populations and those entering a new country as a refugee [38,39]. In contrast, some women felt that being active promoted their psychological well-being. Cultural competence and sensitivity is essential in these circumstances, however, it is necessary that health professionals establish the link between trauma and health and wellbeing for these populations, and clearly outline the psychological and physical benefits associated with preventive health measures such as physical activity. Additionally, Bosnian women also recognised prayer as a form of physical activity due to the up and down movements and rising and lowering of arms during each visit to temple. It may be useful to also build awareness around the physical benefits that may be associated with this religious practice.

Arabic speaking participants indicated that public modesty was a barrier to engaging in physical activity outside of their home due to their religious beliefs and practices. Many women of Muslim faith interpret scriptures of the Qur'an as prohibiting physical activity participation [40], as this is seen to conflict with their family responsibilities [14]. These women feel underpinned by their 'ethic of care' to their children and other family members and believe that taking time out for themselves to engage in physical activities would signify that they were neglecting their role of mother and family caregiver [41,42]. Developing programs in an environment that is mother-child friendly, where mothers can participate in activities with their children, would provide mothers with the opportunity to benefit from physical activity while still upholding their 'ethic of care' [41].

Furthermore, cultural modesty in the form of acceptable dress was also a barrier for these women. Thus, appropriate adjustments may need to be made to activities, activity facilities and activity session times, creating suitable alternatives for these women that will allow them to either participate in their cultural dress or in an environment which is appropriate if they are not in their traditional dress. Recommendations such as holding women-only classes and maintaining closed off sections of the gym or facility for women only should be considered as ways to address these barriers. However, these are only short term solutions. For long term effects, health professionals must work towards empowering these women by placing them at the centre of program development and encouraging them to take lead roles in development and implementation throughout their own communities so they can make changes, develop programs and embark on new initiatives that are meaningful and suitable to them and other members of their CALD group [18,43].

Surprisingly, the Filipino women were the only participants that indicated that changes in socioeconomic status upon migration to Australia limited them from being as active as they were when living in the Philippines. They describe how in the Philippines many of them had relatives to help with childminding as well as housekeepers to deal with domestic duties such as cleaning and preparing meals. Now living in Australia, these women have greater financial restrictions and no longer have the luxury of this type of support. However, many of the women indicated that they had close relationships with other Filipino women in their community, thus it may be worthwhile exploring the possibility of shared childminding and alternate participation times amongst the women. It may also be worthwhile to explore alternative modes of physical activity such as occupational physical activity [44] for these women, given that many of them work fulltime. Research has revealed a numbers of ways in which physical activity can be incorporated into a working day, including lunchtime walks, using the stairs instead of the lifts, and organising physical activity team challenges with other workmates [42,45,46].

Lack of social support and social isolation were specifically reported by the Sudanese women. Commonly revealed as a barrier in many CALD groups [31], researchers have reiterated the importance of developing a social network both within the same culture, and with those from other cultures [47,48]. Furthermore, undertaking group physical activity with others from your social network, or joining a physical activity group with new members, has been reported to positively influence physical activity behaviours, while providing motivational and emotional support and addressing social isolation [49,50]. This strategy may be particularly useful in the initial stages of migration and resettlement, where social isolation is most evident [51].

The Sudanese women also indicated that due to a lack of understanding within Australia of specific Sudanese cultural activities, they were reluctant to engage in such traditional activities. This resonates with previous research suggesting that a lack of self-efficacy can restrict women from being physically active [33,52], and highlights the importance in promoting and encouraging activities in which CALD communities have mastered, enjoy undertaking and feel comfortable performing. Adapting new and unique strategies and initiatives is an asset to promoting physical activity amongst CALD populations. Again, this highlights the importance of cultural competence, sensitivity and the acknowledgement of cultural diversity [18,43]. Additionally, Sudanese women and health professionals should work together to develop new initiatives which include undertaking physical activity with their children. These activities could incorporate the traditional activities they once undertook with their children in Sudan and new initiatives they have learnt since migrating to Australia. This may also open the door to an integrated mother-child program which would include Sudanese participants as well as women and children from different cultures, including Australian-born participants. This type of program would promote physical activity for mothers and their children as well as provide an outlet for social engagement.

### Strengths and Limitations

A major strength of this study is the sample size and diversity, which included women from four different CALD groups. More importantly, our study included three focus groups for each of the four CALD groups (Bosnian, Arabic speaking, Filipino, Sudanese), allowing for a more in-depth examination of the topic or area of study. As a standard protocol for focus group research, it is recommended that three focus groups for each group represented is conducted in order to reach data saturatation and provide a deeper understanding of the issues or topic [13,53]. However, it is important to note that the participants in this study are not considered representative of all adults in their CALD group.

Although this study has made a significant contribution to the literature pertaining to physical activity in CALD groups throughout Australia, the inclusion of only four different CALD groups also becomes a limitation as there are a significant number of other CALD groups in Australia. Given that CALD groups vary in many respects (e.g. culture, religion, language, socioeconomic status, education, employment, etc.) future research should extend to other CALD groups. Furthermore, the sample was limited to CALD women living in Sydney, Melbourne, and Brisbane. Although, it has been documented that the capital cities and surrounding areas of these states have the greatest porportion of CALD populations [20], there are other areas in South Australia, Western Australia and particular rural areas of Australia which are witnessing a rapid growth in CALD populations through international and inter-state migration [20]. Future research should focus on large representative sampling, throughout all states in Australia to clearly establish a national profile regarding the physical activity behaviours of CALD populations, as well as provide a comparison of similarities and differences across multiple sites and multiple CALD groups. This sampling should include more of the existing groups used in this study (Bosnian, Arabic speaking, Filipino, Sudanese), a variety of other CALD groups, groups including both CALD men and women, and groups including CALD individuals from varying age groups. This sampling could also be extended to CALD groups from other countries, providing camparative data on an international level.

## Conclusions

The over-arching purpose of this study was to examine the socio-cultural influences on the physical activity behaviours of CALD women living in Australia by identifing the barriers, constraints and possible enablers to physical activity participation for this population. Common themes emerged from the data, however it is the ethnic-specific themes that have made a significant contribution to the research literature. Ethnic-specific themes indicated that post-war trauma, religious beliefs and obligations, socio-economic status, social isolation and the acceptance of traditional cultural activities, greatly influenced the physical activity behaviours of Bosnian, Arabic speaking, Filipino and Sudanese women living in communities throughout Australia. Implications for future research and practice can be drawn from these findings.

## Declaration of Competing interests

The authors declare that they have no competing interests.

## Authors' contributions

CMC was a Chief Investigator on the study. She designed the study, collected and analysed data, wrote the first draft of the paper and lead the critical review and revision of the paper. GSK was a Chief Investigator on the study. He contributed to the study design, interpretation of the data, and review of the paper. RT was the study researcher. She assisted with the collection and analysis of data, and contributed to the drafting and critical reviewing of the paper. WKM was also a Chief Investigator on the study. He contributed to the study design, interpretation of data, and review of the paper. All authors have read and approved the final manuscript.

## Pre-publication history

The pre-publication history for this paper can be accessed here:

http://www.biomedcentral.com/1471-2458/11/26/prepub
